# Experiences of patients with cancer at health care facilities in Japan: results from a nationwide survey

**DOI:** 10.1186/s12913-021-07184-8

**Published:** 2021-10-29

**Authors:** Tomone Watanabe, Yuichi Ichinose, Mei Matsuki, Takafumi Wakita, Tsutomu Toida, Masato Masuda, Takahiro Higashi

**Affiliations:** 1 Division of Health Services Research, Center for Cancer Control and Information Services, National Cancer Center, Tokyo, Japan; 2 Department of Cancer Health Services Research, Graduate School of Medicine, the University of Tokyo, Tokyo, Japan; 3 Department of Respiratory Medicine, the University of Tokyo, Tokyo, Japan; 4 Department of Social Psychology, Kansai University, Osaka, Japan; 5 Department of Economics, Dokkyo University, Saitama, Japan; 6 University of Ryukyus Hospital Cancer Center, Okinawa, Japan

**Keywords:** Patients with cancer, Patient experience survey, Younger patients, Rare cancer

## Abstract

**Background:**

To elucidate the experience of patients with cancer from diagnosis to early survivorship in Japan using a nationwide questionnaire survey, and to inform the current progress of the cancer control programs.

**Methods:**

The survey was sent to a representative sample of adult patients with cancer identified from the national database of hospital-based cancer registries. The patients’ responses were compared across three groups: patients with rare cancers, patients aged < 40 years, and patients with non-rare cancers aged ≥40 years.

**Results:**

Of 20,488 patients invited to participate in the survey, 8935 (43.6%) responded. Respondents reported an average score of 7.9 out of 10 on global ratings of care. Patients with rare cancers experienced a longer time to diagnosis but the shortest time from diagnosis to first treatment (*p* < 0.05). Patients aged < 40 years rated worse for the majority of the survey items, especially on items that related to communication with medical staff and items referring to early survivorship.

**Conclusion:**

The care experienced by patients with cancer in Japan varies on the basis of age group and cancer type. Efforts should be directed to ensuring prompt access to diagnostic facilities for patients with rare cancers and providing sufficient support to younger patients.

**Supplementary Information:**

The online version contains supplementary material available at 10.1186/s12913-021-07184-8.

## Background

Patient-centered care has garnered increasing attention in recent years. Health care decisions and quality measurements now focus on individual’s specific health needs and desired health outcomes [1]. Patient-centeredness is also important in formulating health care policy. Because a patient’s life is not only about treatment, the realm of an effective health care policy should also incorporate the social aspects of patients’ lives, i.e., access to health information, financial burden due to disease, social isolation or caregiver burden [2]. To this end, the Japan Cancer Control Plan sets out holistic cancer control measures based on the Cancer Control Act [3]. When the Cancer Control Plan was revised in 2012, it explicitly outlined the importance of policy evaluation based on patients’ perspectives [4]. The government then commissioned the National Cancer Center to conduct the first nationwide Cancer Patient Experience Survey to help incorporate patients’ experiences into the policy evaluation process in 2014 [5].

A patient experience survey is a powerful tool used in assessing the process of care delivery, as well as voicing and including patients’ opinions in the policy-making process. Using survey information, countries can establish inclusive and effective nationwide policies [6, 7]. The survey was designed to evaluate patients’ experience regarding various aspects of disease trajectories, such as their experiences with diagnosis, second opinion, fertility preservation, communication with medical professionals, utilizing existing resources, and information provision, from cancer onset to early survivorship. In 2017, the government issued the third revision of the Cancer Control Plan and administered a second survey in 2018. The second survey was modified to include additional aspects of cancer care stipulated in the new policy such as supporting AYA patients, promoting patient-centered care, promoting team-based care, and living with dignity, so as to improve its validity and reliability. However, thus far, the detailed results and analysis from this survey have not been published.

This study sought to elucidate the experience of patients with cancer during and after the treatment they received at hospitals in Japan in 2016 by assessing responses to the second Cancer Patient Experience Survey by comparing the results of patients in three groups: patients with rare cancer, younger patients, and other patients with non-rare cancers aged ≥40 years. The results are expected to provide helpful information to address the needs of patients with cancer, as well as to evaluate the working cancer control plan.

## Methods

### Hospital-based cancer registry

We selected a representative sample of adult patients diagnosed with cancer in 2016 (from January 1st to December 31st) who first commenced their treatment at hospitals that operated hospital-based cancer registries (HBCRs). The HBCR is a cancer incidence reporting system for all designated cancer care hospitals (DCCHs) and many nondesignated hospitals that play similar roles as DCCHs in their local communities/regions. In reference to the population-based cancer registry in Japan, 70–80% of all patients with cancer in Japan are included in the HBCR [8]. HBCR data tend to contain information on a greater number of younger patients and less common cancers (e.g., cancers of the head and neck) because these patients are more likely to be treated at DCCHs where specialists on these rarer cancers practice [9]. We chose the HBCR for our survey because it was the largest patient registry available in the country at the time of the study. The HBCR data contain the following information about all newly diagnosed patients at a hospital: basic information (e.g., hospital names, sex, and date of birth), tumor information (e.g., clinical and pathological stages, tumor-node-metastasis classification, tumor location, and histopathological findings based on the International Classification of Diseases Oncology third edition [ICD-O-3.1]). Each year, the data from the HBCR are submitted to the National Cancer Center.

### Sample selection

We employed a two-stage sampling process. We first selected hospitals. On the basis of a hospital’s designation status, hospitals were classified as prefectural DCCHs (one to two per prefecture), community DCCHs (typically one per community), semi-DCCHs (for rural communities that do not have hospitals that meet all the requirements), and other hospitals. We invited all 53 prefectural DCCHs to participate, as well as 2 randomly selected community DCCHs in each 47 prefecture, 10 randomly selected semi-DCCHs, and 20 other hospitals across Japan, forming a total sample of 177 hospitals. The probability of hospitals being chosen within the categories was proportional to the number of patients with cancer in the hospitals; alternatively, hospitals with a greater number of patients were more likely to be selected as samples. We invited other hospitals of the same category to participate when the selected hospitals could not participate.

The second stage involved patient selection. Patients from the selected hospitals were considered eligible for participation if they were 19 years old or older at the time of diagnosis. Patients with noninvasive disease only or those who did not know their cancer diagnoses were ineligible for inclusion. Patients were then stratified into three groups: those with rare cancer (see Additional file 1; Appendix 1), as defined by European RARECARENet classifications [10] (Group A); younger patients, defined as those aged < 40 years at diagnosis [11, 12] (Group B); and all other patients with non-rare cancers who are 40 years old or above (Group C). Selection for Group A patients preceded patient selection for other groups. For comparative purposes, we also included a maximum of five cancer-free patients from the hospitals. An additional maximum of 20 patients were sampled from each hospital for two additional analyses. A maximum of 10 patients were sampled to test the effect of asking for permission to obtain information from treating hospitals. These patients were informed that the validity of their responses would be assessed (e.g., stage information and types of cancer) in light of HBCR prior to taking the survey. Furthermore, a maximum of 10 patients who were diagnosed with advanced diseases in 2013 instead of 2016 were selected to conduct follow-up on long-term survivors. However, because these patients were not selected for the main purpose, they were not included in this study.

From each hospital, a total of 100 patients (15 from Group A, 15 from Group B, and 70 from Group C) were randomly selected to participate in the survey. The sample size was mainly determined by the budget to be 100 patients per hospitals. Assuming 50% response rate, we calculated the maximum standard errors for the proportion estimates to be up to 7% at the hospital level (50 samples), and up to 4% at the prefecture level (150 samples with three hospitals per prefecture). An initial list of patients was sent to each hospital for a final review. Some patients were then excluded from each hospital for other reasons, such as having no informed consent despite not being documented in the registry. In the event that some patients were unable to participate, we selected other patients in the same category to make up for the loss. After finalizing the list of participants, we sent the survey to a total of 100 patients with cancer from each hospital between January and July 2019. We allowed proxy reporting in case patients were unable to respond to the survey because we intended to report comprehensive experiences of all patients who underwent cancer treatment and omitting proxy reporting would cause the systematic exclusion of certain groups of patients who could not respond to the survey.

### Questionnaire

A questionnaire to evaluate cancer control policies and programs was developed in 2015 for the first Cancer Patient Experience Survey based on focus group discussions with patients with cancer. The focus group discussions provided information on specific aspects of patient experience, which included the following dimensions: optimal care provision, access to correct information and consultation, the financial impact of a cancer diagnosis, social isolation, and caregiver burden. The detailed process composing the questionnaire is reported elsewhere [13]. The questionnaire was developed to cover these dimensions in a time series order: cancer diagnosis, choosing treatment, receiving treatment, and social life during and after treatment. It posed questions in a chronological order related to the periods before, during, and after treatment. This questionnaire was subsequently updated based on revisions made to the Cancer Control Plan. Seven questions on patient-centered care, five questions on younger patients, four questions on team-based care, three questions on-the-job and palliative care, two questions on living with dignity, patient support and providing information, and cancer education were added during this revision. Before finalizing the questionnaire, we conducted cognitive interviews with 10 patients to assess whether patients understood the questions as intended. The questionnaire is included as a supplementary document (see Additional file 1; Appendix 2).

### Data analysis

We performed a weighted analysis to adjust for the sampling probability and nonresponse to ensure an adequate representation of the target population. We then developed a design weight based on our sampling method. The weighted results were displayed as corrected values for all aggregated results.

Patient demographic data such as age, sex, and information on their cancers, along with responses to each question, were collected. As regards the patient categories, the three selected groups (A: rare cancer, B: younger [age 19–39 years], and C: others) were chosen because patients with rare cancer and younger patients are described as an underrepresented population that is in greater need in the Cancer Control Plan. We calculated average scores and proportion of positive responses for all patients and for each group and compared them using the chi-square test. We obtained the percentage of positive responses from the questionnaire: from the five Likert-scale responses, “Agree strongly” and “Agree” were treated as positive responses. “Agree somewhat” was categorized as a nonpositive response to avoid the ceiling effect. For Q17, the percentage excluding those who choose “None of the above happened” was calculated. Likewise, for Q27, the percentage excluding those who chose “I did not experience any of the above” was calculated. For Q31 and Q32, the responses “very familiar” and “somewhat familiar” were treated as positive responses. The percentage of other responses were considered self-explanatory. If the test using the three groups was significant, we then compared two groups, Group A vs. Group C and Group B vs. Group C. We used Group C as a comparison group because we aimed to elucidate the features of the other two groups by considering patients with cancer whose proportion was the largest in the population. The t-test was employed when analyzing continuous variables such as age. The weighted average of global ratings of care was calculated, as well as the weighted percentage of respondents. A *p*-value of < 0.05 was considered statistically significant. The analysis weights were developed using R version 3.6.1, and all analyses were performed on Stata version 15.1 (StataCorp LP, College Station, TX, USA).

### Ethical considerations

Written consent was obtained from each patient prior to completing the questionnaire. This study protocol was approved by the Institutional Review Board of the National Cancer Center, Japan (IRB number 2018–218). All methods were performed in accordance with the relevant guidelines and regulations.

## Results

### Patient characteristics

A total of 20,488 patients across 166 hospitals were invited to participate in the survey, of which 8935 (43.6%) responded. Among the respondents, a total of 1855 patients were selected for other purpose than the main analysis, and thus excluded from the analysis, leaving a total of 7080 respondents for the final analysis.

The demographic characteristics of the 7080 participants are presented in Table 1 (797 from Group A, 705 from Group B, and 5574 from Group C). Because we grouped patients sequentially from Group A to C, 6.8% of patients in Group A (rare cancers) were younger than 40 years old. We also confirmed that the proportion of patients only varied by a maximum of 2% when these patients were recategorized into Group B; therefore, we retained the results of our original classification. After applying weights, the patient characteristics resembled the HBCR data. Study participants were more likely to be men (53.3%), with a mean age of 69.4 (SD = 12.3). The self-reported stage was mainly (28.3%) stage I cancer.

**Table 1:** Patient demographics

		All	% (corrected^a^)	Rare cancer (Group A)	% (corrected^a^)	Younger (Group B)	% (corrected^a^)	Non-rare cancer age ≥ 40 (Group C)	% (corrected^a^)
**N**		7080	11.3 (5.0)	797	11.3 (5.0)	709	10.0 (2.4)	5574	78.7 (92.6)
**Sex**	Male	3688	52.1 (53.3)	477	59.8 (59.9)	128	18.1 (16.6)	3083	55.3 (53.9)
	Female	3368	47.6 (46.2)	318	39.9 (40.0)	581	81.9 (83.4)	2469	44.3 (45.6)
	No response	24	0.3 (0.5)	2	0.3 (0.1)	0	0.0	22	0.4 (0.6)
**Age**^b^	Average (SD)	66.4 (15.3)	69.4 (12.3)	65.8 (15.6)	66.2 (22.6)	36.9 (4.6)	36.8 (9.2)	70.6 (11.1)	70.5 (10.1)
	Median (IQR)	70 (58,77)	71 (63,78)	69 (56,77)	68 (58,77)	38 (35,40)	38 (35,40)	71 (64,78)	71 (64,78)
**Respondent**	Patient	5583	78.9 (78.4)	576	72.3 (70.6)	667	94.1 (92.8)	4340	77.9 (78.5)
	Family	1449	20.5 (20.9)	215	27.0 (27.3)	41	5.8 (7.2)	1193	21.4 (20.9)
	Other	13	0.2 (0.2)	4	0.5 (2.0)	1	0.1 (0.0)	8	0.1 (0.0)
	No response	35	0.5 (0.5)	2	0.3 (0.1)	0	0.0	33	0.6 (0.6)
**Self-reported stage**	0	381	5.4 (5.9)	33	4.1 (3.7)	29	4.1 (4.7)	319	5.7 (6.0)
	I	1960	27.7 (28.3)	135	16.9 (17.9)	239	33.7 (34.1)	1586	28.5 (28.7)
	II	1181	16.7 (17.1)	91	11.4 (10.7)	160	22.6 (24.0)	930	16.7 (17.3)
	III	899	12.7 (13.5)	90	11.3 (10.0)	65	9.2 (9.3)	744	13.3 (13.8)
	IV	1099	15.5 (15.2)	162	20.3 (22.9)	61	8.6 (7.7)	876	15.7 (14.9)
	Unknown	1339	18.9 (17.1)	267	33.5 (31.9)	136	19.2 (18.2)	936	16.8 (16.3)
	No response	221	3.1 (2.9)	19	2.4 (3.0)	19	2.7 (2.0)	183	3.3 (3.0)
**Self-reported cancer type**	Breast	959	13.5 (13.7)	13	1.6 (1.6)	216	30.5 (35.2)	730	13.1 (13.8)
	Colon	1023	14.4 (17.1)	35	4.4 (5.6)	55	7.8 (7.7)	933	16.7 (18.0)
	Stomach	986	13.9 (16.3)	25	3.1 (6.0)	31	4.4 (7.6)	930	16.7 (17.0)
	Lung	871	12.3 (13.4)	37	4.6 (5.1)	30	4.2 (3.7)	804	14.4 (14.1)
	Liver	289	4.1 (4.7)	13	1.6 (1.3)	13	1.8 (1.1)	263	4.7 (5.0)
	Prostate	737	10.4 (11.0)	19	2.4 (3.3)	13	1.8 (1.1)	718	12.9 (11.7)
	Cervix, Uterus	414	5.8 (4.6)	27	3.4 (3.1)	122	17.2 (14.0)	265	4.8 (4.5)
	Ovary	151	2.1 (2.0)	6	0.8 (0.4)	29	4.1 (2.5)	116	2.1 (2.0)
	Esophagus	227	3.2 (3.7)	8	1.0 (2.3)	4	0.6 (0.4)	215	3.9 (3.8)
	Spleen	177	2.5 (2.6)	1	0.1 (0.0)	3	0.4 (0.2)	173	3.1 (2.8)
	Oral	382	5.4 (3.7)	228	28.6 (30.6)	7	1.0 (1.2)	147	2.6 (2.4)
	Thyroid	205	2.9 (2.0)	5	0.6 (0.4)	81	11.4 (8.4)	119	2.1 (1.9)
	Lymphoma, leukemia	515	7.3 (6.7)	83	10.4 (8.0)	97	13.7 (13.8)	335	6.0 (6.5)
	Bone, soft tissue sarcoma	100	1.4 (1.0)	58	7.3 (8.1)	4	0.6 (0.8)	38	0.7 (0.6)
	Brain	102	1.4 (1.0)	54	6.8 (7.2)	3	0.4 (0.3)	45	0.8 (0.7)
	Bladder	178	2.5 (2.8)	9	1.1 (1.7)	2	0.3 (0.2)	167	3.0 (2.9)
	Testicle	50	0.7 (0.3)	47	5.9 (4.7)	13	1.8 (1.1)	3	0.1 (0.1)
	Unknown primary	40	0.6 (0.7)	10	1.3 (1.8)	5	0.7 (0.8)	25	0.4 (0.6)
	Other	645	9.1 (7.8)	218	27.4 (27.3)	28	3.9 (6.0)	399	7.2 (6.8)
	No response	147	2.1 (2.0)	20	2.5 (2.4)	13	1.8 (1.1)	114	2.0 (2.0)

*Abbreviations*: *AYA* Adolescent and young adult

^a^Design weight was developed based on the sampling method, and the weight is the inverse of the sampling fraction

^b^Age at the time of response

When the three groups were compared, 80% of all respondents in Group B were female, and the number of patients with uterine and/or breast cancer accounted for 49.2% of all respondents as compared with 18.3% in Group C.

### Global ratings of care

The average global ratings of care were 7.9 out of 10. Among all respondents, 79.0 and 77.3% were satisfied with treatment selection and treatment experience, respectively. In addition, 78.7% of respondents perceived that their providers had sufficient level of expertise in their cancer. The global ratings of care for each group were 8.0 for Group A, 7.8 for Group B, and 7.9 for Group C (*p* = 096).

Table 2 shows the results of the comparison between the three groups based on the chronological period (before, during, or after treatment). Of all 52 items, the response scores of Group A significantly differed from those of Group C for eight items. In contrast, the scores for 25 items were statistically different between Group B and Group C.

**Table 2:** Survey results^a^ (Q1–Q7 were demographic questions)

Items	All	Rare cancer (Group A)	Younger (Group B)	Non-rare cancer age ≥ 40 (Group C)	*p*-value
**Before treatment**					
Time from the first consultation to diagnosis was < 1 month (Q8)	71.5%	66.4%	66.2%	71.9%	A − C: *p* = 0.03 B − C: *p* = 0.07
Time from diagnosis to the first treatment was < 1 month (Q9)	62.2%	72.3%	52.5%	62.0%	A − C: *p* < 0.01 B − C: *p* = 0.01
I was able to talk about cancer or life as a cancer patient with someone after diagnosis (Q10)	76.3%	77.9%	89.0%	75.9%	A − C: *p* = 0.41 B − C: *p* < 0.01
My doctor advised me of the possibility of obtaining a second opinion (Q11)	34.9%	35.2%	27.9%	35.1%	A − C: *p* = 0.98 B − C: *p* = 0.01
I received a second opinion (Q12)	19.5%	21.9%	19.4%	19.4%	*p* = 0.54
I received enough information from medical staff before making treatment decision (Q13–1)	75.0%	75.7%	65.4%	75.2%	A − C: *p* = 0.77 B − C: *p* = 0.02
I am content with my choice of treatment (Q13–2)	79.0%	81.4%	76.1%	78.9%	*p* = 0.45
I was informed about the possibility of infertility before treatment (age < 40) (Q14)	52.0%				
I took some actions for fertility preservation (age < 40) (Q15)	8.9%				
I changed or discontinued treatment due to financial reasons (Q16)	4.9%	4.2%	11.1%	4.8%	A − C: *p* = 0.55 B − C: *p* = 0.03
I altered my financial plans or sought assistance from others to cover my medical expenses (Q17)	26.9%	28.4%	53.1%	26.1%	A − C: *p* = 0.47 B − C: *p* < 0.01
**During treatment**					
I received enough information about the treatment schedule (Q18–1)	75.1%	75.7%	72.0%	75.1%	*p* = 0.55
I was able to anticipate the likely side effects of treatment (Q18–2)	61.9%	63.6%	58.4%	62.0%	*p* = 0.66
I had detailed discussions with medical staff about my treatment (Q18–3)	67.5%	72.6%	57.8%	67.5%	A − C: *p* = 0.06 B − C: *p* = 0.04
Medical staff listened and tried to understand my concerns (Q18–4)	71.9%	79.7%	71.6%	71.4%	A − C: *p* < 0.01 B − C: *p* = 0.98
My wishes regarding the treatment were respected (Q18–5)	73.9%	77.3%	75.4%	73.7%	*p* = 0.33
Medical staff responded to my pain or discomfort promptly (Q18–6)	75.0%	79.6%	72.0%	74.8%	*p* = 0.07
Relevant information was shared among medical staff (Q18–7)	69.1%	72.0%	63.7%	69.0%	A − C: *p* = 0.26 B − C: *p* = 0.05
I received treatment from a doctor with expertise (Q18–8)	78.7%	80.0%	85.7%	78.4%	A − C: *p* = 0.53 B − C: p = 0.03
I felt comfortable talking to the medical staff besides my doctor (Q18–9)	48.8%	53.7%	52.2%	48.5%	*p* = 0.32
I am satisfied with treatment I received (Q18–10)	77.3%	77.5%	83.5%	77.1%	A − C: *p* = 0.85 B − C: *p* = 0.04
Medical staff offered enough information regarding aspects of daily life while admitted (applicable patients only) (Q18–11)	71.1%	75.7%	73.5%	70.7%	*p* = 0.20
I visited a referral hospital without any trouble (applicable patients only) (Q18–12)	82.5%	80.8%	79.5%	82.7%	*p* = 0.79
I was transferred to my preferred hospital (applicable patients only) (Q18–13)	79.2%	78.3%	75.5%	79.4%	*p* = 0.72
I was asked on every consultation if I had pain during or after treatment (Q19)	65.3%	71.1%	64.8%	65.0%	*p* = 0.10
I was able to discuss my concerns about the changes in appearance due to treatment (Q20)	28.3%	32.0%	46.3%	27.6%	A − C: *p* = 0.08 B − C: *p* < 0.01
Overall experience from diagnosis through to treatment (0–10, average) (Q21)	7.9	8.0	7.8	7.9	*p* = 0.96
I was engaged in paid employment at the time of diagnosis (Q22)	44.2%	50.0%	81.7%	42.9%	A − C: *p* = 0.02 B − C: *p* < 0.01
I told my colleagues about my diagnosis (applicable patients only) (Q23)	81.0%	85.8%	95.3%	79.9%	A − C: *p* = 0.13 B − C: *p* < 0.01
My colleagues considered and managed the situation so that I could keep working while receiving treatment (applicable patients only) (Q24)	65.0%	69.8%	68.6%	64.5%	*p* = 0.15
I utilized existing resources to balance my treatment and work (applicable patients only) (Q25)	36.1%	34.0%	51.4%	35.5%	A − C: *p* = 0.78 B − C: *p* < 0.01
I received some advice from the medical staff about continuing to work (applicable patients only) (Q26)	39.5%	36.6%	54.9%	38.9%	A − C: *p* = 0.61 B − C: *p* < 0.01
I resigned or closed business due to treatment (applicable patients only) (Q27–1-1)	19.8%	19.6%	20.5%	19.8%	*p* = 0.96
I took leave of absence but did not resign or close business (applicable patients only) (Q27–1-2)	54.2%	54.3%	57.1%	54.0%	*p* = 0.72
**After treatment (items related to survivorship)**					
I feel that cancer treatment for the general public has improved compared to a few years ago (Q28–1)	75.6%	73.5%	69.5%	75.8%	A − C: *p* = 0.43 B − C: *p* = 0.03
I feel that there is sufficient support, services, and places for cancer patients and their families to discuss their concerns about cancer (Q28–2)	47.7%	46.1%	39.5%	48.0%	A − C: *p* = 0.51 B − C: *p* < 0.01
I am aware of cancer counseling and support centers (Q29)	66.4%	63.3%	67.5%	66.5%	*p* = 0.35
I am aware of peer support (Q30)	27.3%	22.3%	19.2%	27.8%	A − C: *p* = 0.05 B − C: *p* < 0.01
I know what clinical trials are (Q31)	39.7%	37.5%	44.3%	39.7%	*p* = 0.40
I am aware of genome-based cancer treatments (Q32)	17.0%	13.5%	16.3%	17.1%	A − C: *p* = 0.04 B − C: *p* = 0.75
I am a burden on my family because of my cancer (patients only) (Q33–1)	47.2%	53.1%	58.1%	46.6%	A − C: *p* = 0.08 B − C: *p* < 0.01
I am a burden on people outside of my family because of my cancer (patients only) (Q33–2)	21.4%	30.2%	40.0%	20.3%	A − C: *p* < 0.01 B − C: *p* < 0.01
I received too much unnecessary attention after my cancer diagnosis (patients only) (Q33–3)	12.3%	13.2%	22.6%	11.9%	A − C: *p* = 0.47 B − C: *p* < 0.01
I feel discriminated against by people outside of my family because I have cancer (patients only) (Q33–4)	5.3%	5.2%	15.4%	4.9%	A − C: *p* = 0.88 B − C: *p* < 0.01
I am able to consult with medical staff when feeling pain or discomfort (patients only) (Q33–5)	46.5%	47.8%	36.2%	46.8%	A − C: *p* = 0.80 B − C: *p* < 0.01
I am able to consult with medical staff when experiencing mental distress (patients only) (Q33–6)	32.8%	33.3%	22.0%	33.1%	A − C: *p* = 0.94 B − C: *p* < 0.01
I am able to go about my daily life now (patients only) (Q33–7)	70.5%	69.2%	66.8%	70.7%	*p* = 0.50
I have sufficient support to relieve my physical pain and mental distress (patients only) (Q34–1)	43.0%	42.1%	37.1%	43.1%	A − C: *p* = 0.80 B − C: *p* = 0.05
I have no physical distress caused by cancer or cancer treatment (patients only) (Q34–2)	55.4%	49.4%	49.8%	56.0%	*p* = 0.25
I have no pain caused by cancer or cancer treatment (patients only) (Q34–3)	71.5%	63.7%	65.6%	72.2%	A − C: *p* = 0.02 B − C: *p* = 0.16
I have no mental distress due to cancer or cancer treatment (patients only) (Q34–4)	62.0%	57.0%	48.9%	62.8%	A − C: *p* = 0.18 B − C: *p* < 0.01
I have no difficulties going about my daily life due to pain and discomfort from cancer or cancer treatment (patients only) (Q34–5)	69.2%	66.5%	59.8%	69.7%	A − C: *p* = 0.48 B − C: *p* < 0.01

*Abbreviations*: *AYA* Adolescent and young adult

^a^Design weight was developed on the basis of the sampling method, and the weight is the inverse of the sampling fraction

These findings suggest that although the global ratings of care remain the same among patients across different groups, care received is experienced differently between the groups.

### Pretreatment experiences

Table 2 presents the overall and group-stratified results of patients’ pretreatment experiences. Approximately 60–70% of patients scored favorably in case of most questions. Of note, however, only a limited proportion reported that their doctors advised them of the possibility of obtaining a second opinion (Q11, 34.9%), and the possibility of infertility due to the treatment (Q14, 52.0% of patients under 40 years old). As for group-stratified results, patients in Group A rated care comparable to patients in Group C, whereas patients in Group B rated care less favorably to patients in Group C. Notable results were observed regarding access time. Patients in Group A experienced a long wait from the first visit to diagnosis (proportion of patients who received a diagnosis in < 1 month, 66.4% vs. 71.9%, *p* = 0.03); however, their time from diagnosis to treatment was shortest (proportion of patients whose treatment began < 1 month from diagnosis, 72.3% vs. 62.0%, *p* < 0.01). More patients in Group A responded negatively to the question about their perception about having received treatment from a doctor who had enough expertise in treating their cancers (4.9% vs. 1.9%, *p* < 0.01). Patients in Group B scored worse on 6/9 items referring to pretreatment, notably in the field of financial impact and information needs.

These findings highlight different areas of support that are in demand from Group A and Group B patients regarding the care they experience before treatment.

### Treatment and posttreatment experiences

We also presented patient experience regarding care provided during and after treatment, that is, items referring to survivorship (see Table 2). Note that only 48.8% felt comfortable talking to the medical staff besides their doctors (Q18–9), 39.5% received advice from the medical staff about continuing work (Q26), 47.7% felt that there was sufficient support to families (Q28–2), 27.7% knew of peer support, 46.5 and 32.8% were able to consult with medical staff in the event of physical pain or mental distress (Q33–5 & 6), and 43.0% felt that they had sufficient support to relieve their physical pain and mental distress (Q34–1). Although patients in Group A rated care more favorably than patients in Group C, patients in Group B rated a majority of items as worse than those in Group C, especially on items referring to their survivorship. In addition, the results show that Group B patients suffered from vocational difficulties more often than patients in other groups. However, fewer patients in Group B had difficulties initiating communication with medical staff (Q13–1, Q18–3, Q33–5, 6). Although Group B patients scored better on some items referring to care during treatment, most items (12/18 items) referring to the posttreatment phase compared unfavorably with those from Group C, and no item was scored better than patients in Group C in this phase.

These findings suggest that care experienced by patients in Group B compares unfavorably especially in the posttreatment phase when the items refer to their early survivorship.

## Discussion

Through a nationwide survey, we evaluated the experience of care and social life of patients with cancer from diagnosis to early survivorship in Japan from 2016 to 2018. Overall, patients favorably rated their care (7.9/10), but perceptions toward care varied across cancer types and age groups. In particular, patients with rare cancers experienced a long delay from the first visit to the diagnosis and were more likely to report having seen a doctor who they did not trust to be an expert in his or her field. In addition, younger patients reported significantly less satisfaction with active communication with medical staff, as well as with items related to their survivorship in post treatment care.

The questions to which patients answered unfavorably give us insights as to where we should improve the care delivery. For example, fewer patients were asked to seek a second opinion. Because many patients may fear that seeking second opinion may offend their doctors [14], such patients should be informed of this option by their doctors. During treatment, most patients did not feel comfortable about communication with medical staff; less than half of patients felt comfortable talking to the medical staff besides their doctors or consult with medical staff about their physical and psychological distress. In addition, a minority of patients felt that they had sufficient support to relieve their physical pain and distress. The difficulty in establishing relationships with medical staff other than doctors may be attributed to the medical system. Excluding doctors, all medical staff usually take shifts, resulting in patients being attended to by different staff at each visit. Because doctors often seem unavailable outside their consultation, team-based care might allow expanded access to care (more hours of coverage, shorter wait times) [15]. We need to construct a system that promotes trusting relationships between various medical staff and patients from an early stage of cancer care so that patients can receive accessible, comprehensive care from any medical personnel. Support to family is also an important aspect of cancer care that needs improvement, given that only 47.7% of patients felt that sufficient support was provided. Because our survey did not contain detailed questions regarding this topic, future research should identify which respondents were in need of family support.

Internationally, surveys regarding cancer patient experience have been conducted in many countries. Although direct comparisons may be difficult, patients express similar concerns across the board. In the experience surveys of English and Japanese patients, global rating of care was 8.8 and 7.9, respectively [16]. As for questions regarding during treatment, 88% patients answered they were treated with respect and dignity in England while 73.9% think that their wishes regarding the treatment were respected in Japan. As for posttreatment, although questions did not cover same areas of care, patients who feel that they have enough care and support from health or social services were very low (21.1%) in England while 43.0% feel they have sufficient support to relieve their physical pain and mental distress in Japan. Because a survey in the United States showed that patients in better condition report better care experiences [17], characterizing the mechanisms of care experiences and level of satisfaction may worth exploring in future research. Adding more international evidence in this field enables us to compare patient experiences under differing healthcare systems. This provides us with a benchmark for measuring the working healthcare system in a country, which may be help us seek areas of improvement in a given healthcare plan.

The delay between the first visit and diagnosis for patients with rare cancers observed in our study is consistent with previous reports. Among many challenges faced by this population, incorrect or late diagnosis has been an issue owing to the difficulty of diagnosis [18]. On top of time from the first consultation to diagnosis, a previous study suggested that time from symptom onset to the first consultation is longer for this population [19]. Because the length of time from diagnosis to the first treatment for patients with rare cancers was the shorter than the other groups analyzed in the current study, once the diagnosis is known, they readily receive treatment. This could suggest the need for timely diagnosis for this population.

Our results suggesting that younger patients with cancer are in greater need of help in many aspects of their disease trajectories corroborate previously published evidence. Similar to previous studies, our study confirms that younger patients have greater unmet needs in the areas of financial challenges, receiving information, and sense of isolation on top of other barriers to health services [20–22]. As for survivorship, a systematic literature review conducted in 2016 highlights the specific needs of younger survivors in four areas, namely, physical well-being, psychological well-being, social well-being, and survivorship care, which also align with the findings of our study [23]. It is also manifested by a study conducted in the U.S. suggesting that AYA patients with cancer reported their quality of life to be much lower than similarly-aged population, and this finding supports the need for specific intervention for this population [24].

Our study highlighted challenges younger patients face in active communication with medical staff and found that, in early survivorship, they feel more isolated, have less trust and satisfaction in existing resources, and experience more physical and mental distress compared with general patients. The findings can be solidified by implications that suggest that although younger patients with cancer have greater resilience while in treatment [25], higher stress levels are predicted in survivorship, especially in the early posttreatment phase [26, 27]. Because their posttreatment needs, both physical and psychosocial, vary over time [28], a time-dependent assessment is vital for this population. Furthermore, although there is a study concluding that female patients tend to report their experiences more unfavorably [29], further research is needed to confirm if the elevated proportion of female patients (with breast and/or uterine cancer) in the group could account for this result. Further analysis should confirm what the detailed factors are that attribute to the level of satisfaction, especially for this population.

This study has some limitations. First, our target population was patients who received care at relatively larger hospitals that have HBCRs. Patients who were treated at small hospitals may have had a different experience. However, the HBCR covers approximately 70–80% of all new cases in Japan, and this is the largest database that can be used for survey purposes, so we believe that our sample population is adequately representative of the entire Japanese cancer patient population. Second, it is possible that hospitals that are relatively worse-off dropped out of the survey. Likewise, unsatisfied patients may have declined to participate in our survey, which could have led to an overestimation of patients’ satisfaction. Nevertheless, respondent characteristics did not differ considerably from those of the target population, suggesting that our sample population accurately reflects the current situation. Third, the results would have been different had we not permitted proxy reporting. However, proxy reporting is considered relatively valid [30] and although it could underestimate patients’ experience [31], coupling it with the higher percentage of proxy reporting among group C patients (7.2% vs. 20.9% between Group B and Group C), the greater need of younger patients will only be enunciated. Fourth, owing to the exploratory nature of the survey, we conducted statistical analysis to compare groups A–C with respect to all items. Because of multiple testing, some statistical significance observed might be due to chance. Finally, the female predominance of younger patients (Group B) may have influenced our findings. However, as breast and cervical cancers are increasingly prevalent in younger patients in Japan [32], we believe that our results are an accurate reflection of the current state of cancer care in Japan. Future surveys should pursue deeper understandings such as drivers of good/poor experience and potential intervention for improvement.

## Conclusions

This study demonstrated that the care experienced by patients within Japan differs across age groups and cancer types. Importantly, our results suggest that ensuring prompt access to health care for patients with rare cancer and supporting younger patients to engage in active communication with medical staff to obtain both physical and psychosocial support, especially in the posttreatment phase, are key to improving patient experience. Through further studies, we need to understand more about drivers of poor experience, undertake efforts to prevent poor experience, and address unmet needs. Future surveys should focus on individual themes and be constructed in a way that enables better understanding of the mechanisms that shape the experiences of this population.

## Supplementary Information

**Additional file 1:** **Appendix 1.** Rare cancer list. **Appendix 2.** Patient Experience Survey. **Appendix 1.** A list of rare cancer used in this study. **Appendix 2.** English translated version of the questionnaire (Patient Experience Survey).

## Data Availability

The datasets generated during and/or analyzed during the current study are available from the corresponding author on request.

## Abbreviations

AYA: Adolescence and young adulthood
DCCH: Designated cancer care hospitals
HBCR: Hospital-based cancer registries
IPOS: Integrated Palliative care Outcome Scale
