# Supplementary material for: Experiences of patients with cancer at health care facilities in Japan: results from a nationwide survey
Source: BMC Health Serv Res. 2021 Oct 29;21:1180. doi: 10.1186/s12913-021-07184-8 (PMC8556904; doi:10.1186/s12913-021-07184-8)
Supplement: Supplementary file 1 — Additional file 1: Appendix 1. Rare cancer list. Appendix 2. Patient Experience Survey. Appendix 1. A list of rare cancer used in this study. Appendix 2. English translated version of the questionnaire (Patient Experience Survey). [file 12913_2021_7184_MOESM1_ESM.docx]

Appendix 1: Rare cancer list*

| Types of tumor | |
| --- | --- |
| 1 | Pleuropulmonary blastoma |
| 2 | Pancreatoblastoma |
| 3 | Odontogenic malignant tumors |
| 4 | Carcinomas of parathyroid gland |
| 5 | Epithelial tumors of middle ear |
| 6 | Carcinomas of pituitary gland |
| 7 | Trophoblastic tumor of placenta |
| 8 | Nephroblastoma |
| 9 | Hepatoblastoma |
| 10 | Kaposi's sarcoma |
| 11 | Malignant melanoma of uvea |
| 12 | Epithelial tumor of trachea |
| 13 | Retinoblastoma |
| 14 | Olfactory neuroblastoma |
| 15 | Histiocytic and dendritic cell neoplasms |
| 16 | Embryonal tumors of cns |
| 17 | Carcinoma of adrenal cortex |
| 18 | Epithelial tumors of eye and adnexa |
| 19 | Neuroblastoma and ganglioneuroblastoma |
| 20 | Epithelial tumors of urethra |
| 21 | Extragonadal germ cell tumors |
| 22 | Non epithelial tumors of ovary |
| 23 | Malignant melanoma of mucosa and extracutaneous |
| 24 | Epithelial tumors of penis |
| 25 | Bone sarcoma |
| 26 | Epithelial tumors of nasopharynx |
| 27 | Epithelial tumors of anal canal |
| 28 | Epithelial tumors of thymus |
| 29 | Gastrointestinal stromal sarcoma |
| 30 | Epithelial tumors of nasal cavity and sinuses |
| 31 | Epithelial tumors of vulva and vagina |
| 32 | Malignant mesothelioma |
| 33 | Adnexal carcinoma of skin |
| 34 | Testicular and paratesticular cancers |
| 35 | Epithelial tumors of major salivary glands and salivary-gland type tumors |
| 36 | Epithelial tumors of small intestine |
| 37 | Malignant skin melanoma |
| 38 | Tumors of central nervous system (cns) |

* Types of rare cancers used in this study were defined by European RARECARENet classifications. Incidence was obtained from 2013-2015 Hospital-Based Cancer Registry, and cancers that were defined in the classification were categorized as rare in an ascending order. Sarcoma, Oral epithelial tumor and Mature T-cell and NK lymphoma were also included.

Appendix 2: Patient Experience Survey

**About yourself/patient**

1. **Do you agree to participate in this survey? (Choose one)**
2. Agree → (Please go to question 2)
3. Disagree → (This is the end of the questionnaire. Thank you very much.)
4. **Sex (Choose one)**

a. Male b. Female

1. **What is your birth year? (Choose Japanese era from a to d, and provide your birth year)**

a. Meiji b. Taisho c. Showa d. Heisei

Year:

1. **What cancer treatments, including treatments for any recurrences, have you received to date? (Choose one)**
2. I have received the following treatments:

Please choose all that apply

a1. Surgery

a2. Endoscope therapy

a3. Chemotherapy (including molecularly targeted therapy/immunotherapy^*^)

a4. Hormone treatment

a5. Radiotherapy

a6. Palliative care

a7. Others ()

1. I have not received any treatment.

**covered by health insurance*

1. **If you have received treatment for two or more cancers, including recurrences, please describe your most recent treatment. (Choose one)**
2. I have finished treatment and regular visits.
3. I have finished treatment and am now having regular visits.
4. I am currently being treated.

I have received the following treatments. (Choose all that apply.)

a1. Surgery

a2. Endoscope therapy

a3. Chemotherapy (including molecularly targeted therapy/immunotherapy^*^)

a4. Hormone treatment

a5. Radiotherapy

a6. Palliative care

a7. Others ()

1. I have not received treatments
2. Others ()

**covered by health insurance*

1. **What kind of cancer (primary tumor*) were you diagnosed with during the last five years? If you received treatment for more than two cancers, or recurrence of a cancer, please answer regarding the most recent cancer or recurrence. (Choose any that apply)**

a. Breast cancer b. Colorectal cancer c. Stomach cancer

d. Lung cancer e. Liver cancer f. Prostate cancer

g. Uterine cancer, cervical cancer h. Ovarian cancer i. Esophageal cancer

j. Pancreatic cancer k. Oral, pharyngeal, larynx cancer l. Thyroid cancer

m. Malignant lymphoma, leukemia n. Bone and soft tissue tumor o. Brain tumor

p. Bladder cancer q. Testicular tumor r. Primary unknown cancer

s. Others ()

*** *Metastasis may occur at sites other than at the original site, but the cancer of the original site is called the primary tumor.*

1. **What stage was the cancer at when you were initially diagnosed? If it was not certain, please choose the nearest stage. If you received more than one diagnosis, please answer in respect to the cancer you were diagnosed with most recently. (Choose one)**
2. Stage 0
3. Stage 1
4. Stage 2
5. Stage 3
6. Stage 4
7. I do not know.

**The following questions are about your pretreatment experiences**

**If you received treatment for more than two cancers, or recurrence of a cancer,**

**please answer regarding the most recent cancer or recurrence.**

1. **How long did it take you to receive a medical diagnosis from the time of (i) your first visit to a doctor because of symptoms you were experiencing or (ii) if the abnormality was in fact first detected during a health check-up with your doctor, from the time of such check-up? (Choose one)**
2. Less than two weeks
3. More than two weeks and less than one month
4. More than one month and less than three months
5. More than three months and less than six months
6. More than six months
7. I do not know
8. **How long did it take to start your first treatment after receiving the diagnosis from your doctor? (Choose one)**
9. I started the treatment before the diagnosis → (please go to question 14)
10. Less than 2 weeks
11. More than 2 weeks and less than 1 month
12. More than 1 month and less than 3 months
13. More than 3 months and less than 6 months
14. More than 6 months
15. No treatment → (Please go to question 16)
16. I do not know
17. **Did you talk about your cancer or your life as a cancer patient with anyone after your diagnosis but before starting your treatment? (Choose one)**
18. I did not need to
19. I needed to, but I did not discuss it with anyone
20. I talked to someone

Who did you talk to (Choose all the people you talked to)

c1. Your doctor c2. Nurse

c3. Medical staff c4. Staff at cancer support center

c5. Family member c6. Friend

c7. Other patient with cancer (including cancer patient group)

c8. Online

c9. Others ()

**If you do not mind, please tell us what you were concerned about in the Additional Comments section on page 19.**

1. **Did your doctor advise you of the possibility of obtaining a second opinion* before you started the treatment? (Choose one)**
2. Yes
3. No

Did you ask for a second opinion? (Choose one)

b1. No

b2. Yes, I or a family member did.

**This is seeking the views or opinion of another doctor in respect to your cancer diagnosis and/or treatment plan*

1. **Did you receive a second opinion? (Choose one)**
2. Yes
3. No
4. I do not know
5. **Please choose one response to each of the statements below.**

|  |  |  |  |  | Disagree | Neither agree nor disagree | Agree somewhat | Agree | Strongly agree |
| --- | --- | --- | --- | --- | --- | --- | --- | --- | --- |
| 1. | I received enough information from the medical staff before making my decision with respect to my cancer treatment. | | | | 1 | 2 | 3 | 4 | 5 |
| 2. | I made a reasonable decision with respect to my cancer treatment given the situation I was in following my diagnosis. | | | | 1 | 2 | 3 | 4 | 5 |

1. **Were you informed about the possibility of infertility (including male infertility) before your first cancer treatment? This question is to be completed by everyone, including those who did not need any such information or explanation. (Choose one)**
2. No

Did you need an explanation? (Choose one)

a1. Yes

a2. No

1. Yes

What kind of information did you receive? (Choose one)

b1. It would not affect your pregnancy or your fertility

b2. There was a risk of infertility and I received information/advice/explanation as to how this risk may be minimized or prevented, including through preservation of sperm/eggs.

b3. There was a risk of infertility, but I received information/advice/an explanation that there was neither a possibility of preventing infertility nor of preserving sperm/eggs.

b4. There was a risk of infertility, but I was not provided with any information/advice/explanation about how this may be prevented or minimized, including through preservation of sperm/eggs.

b5. I do not know.

1. I do not know
2. **Owing to the risk of infertility, did you take any action to prevent or minimize the risk of infertility (including changing the treatment or preserving your sperm/eggs)? (Choose one)**

a. Yes b. No c. I do not know

**The following questions are about your treatment**

1. **Have you ever changed or discontinued your cancer treatment owing to financial reasons? (Choose one)**
2. No
3. Yes

What kind of treatment would you have liked to have received had you not had financial constraints? (Choose one from below)

b1. Treatment not covered by health insurance (including advanced medical treatment)

b2. Treatment covered by health insurance

b3. I do not know

1. **Have you ever done or experienced any of the following to pay your medical costs? (Choose any that apply)**
2. Reduced spending on food and clothes
3. Visited the clinic less frequently or cancelled planned visits
4. Asked a doctor to prescribe cheaper medication or treatment
5. Took reduced treatment or dosage of medication without a doctor’s recommendation to do so
6. Used your savings to pay for the costs
7. Family members took on additional hours or additional jobs
8. Received financial support from relatives or a third party (including taking on additional debt)
9. Sold a car, a house or any other real estate, or moved to a new home
10. A family member changed university or gave up university
11. Others ()
12. None of the above happened
13. I do not know
14. **Please choose one response to each of the statements below.**

|  |  |  |  |  | Disagree | Neither agree nor disagree | Agree somewhat | Agree | Agree strongly |
| --- | --- | --- | --- | --- | --- | --- | --- | --- | --- |
| 1. | I was provided with enough information about the treatment schedule. | | | | 1 | 2 | 3 | 4 | 5 |
| 2. | From all the information given to me by the medical staff, I was able to imagine the likely side effects of the treatment. | | | | 1 | 2 | 3 | 4 | 5 |
| 3. | I had detailed discussions with the medical staff about my treatment. | | | | 1 | 2 | 3 | 4 | 5 |
| 4. | The medical staff listened to me and tried to understand my concerns. | | | | 1 | 2 | 3 | 4 | 5 |
| 5. | My wishes regarding the treatment were respected. | | | | 1 | 2 | 3 | 4 | 5 |
| 6. | The medical staff responded to my pain or discomfort. | | | | 1 | 2 | 3 | 4 | 5 |
| 7. | Relevant information about me was shared well among the medical staff involved in my treatment. | | | | 1 | 2 | 3 | 4 | 5 |
| 8. | I received treatment from a doctor that specializes in cancer treatment. | | | | 1 | 2 | 3 | 4 | 5 |
| 9. | I felt comfortable when talking to the medical staff (besides the attending physician) involved in my treatment. | | | | 1 | 2 | 3 | 4 | 5 |
| 10. | I am satisfied with the treatment I received. | | | | 1 | 2 | 3 | 4 | 5 |
| **Were you admitted to hospital during your cancer treatment? (Choose one)** a. No → (Please go to sub-question 12)  b. Yes → (Please go to sub-question 11) | | | | | | | | | |
| 11. | From the time of receiving the first treatment until I was discharged from hospital, I was able to obtain sufficient information from the medical staff regarding aspects of my daily life (e.g., diet, symptoms to note, etc.). | | | | 1 | 2 | 3 | 4 | 5 |
| **Since starting your cancer treatment, have you been transferred to another medical institution? (Choose one)**  a. Yes → (Please go to question 19)  b. No → (Please go to sub-question 12) | | | | | | | | | |
| 12. | I was able to visit the referred medical institution without any problems. | | | | 1 | 2 | 3 | 4 | 5 |
| 13. | I was able to be transferred to the medical institution I wanted | | | | 1 | 2 | 3 | 4 | 5 |

1. **Whenever you were undergoing the cancer treatment, or after such treatment, were you asked if you had any pain? (Choose one)**
2. Yes
3. No
4. I do not know
5. **Were you able to discuss with anyone your concerns about the changes in your appearance (e.g., hair loss or skin problems) due to the cancer treatment? (Choose one)**
6. I did not need to
7. I did not know if I needed to talk
8. I needed to talk to someone but I could not
9. Yes, I was able to
10. I do not know
11. **On a scale of 0–10, how would you rate your overall experience from diagnosis through to cancer treatment? (Choose one number on the following scale, where 0 is the worst and 10 is the best)**

0 1 2 3 4 5 6 7 8 9 10

(Worst) (Best)

**If you have any comments, please tell us in the Additional Comments section on page 19.**

**The following questions are about your job**

1. **At the time of your cancer diagnosis, were you engaged in paid employment? (Choose one)**
2. Yes

What was the type of employment? (Choose one)

a1. Full time employee a2. Self-employed

a3. Contractor a4. Part-time employee

a5. Temporary staff/agency worker a6. Other ()

1. No

**If you have not worked since you were diagnosed with cancer,**

**please go to question 28.**

1. **Did you tell any of your colleagues that you were diagnosed with cancer? (Choose one)**
2. Yes

Who did you talk to? (Choose any that apply)

a1. Your managers or anyone more senior in the company

a2. Your peers at the office

a3. Your subordinates

a4. Human resources department

a5. Medical staff at the office

a6. Labor union

a7. Workplace counselor

a8. Others ()

1. No
2. I do not know
3. **Which of the following best describes your situation (Choose one)**

|  |  |  |  |  | Disagree | Neither agree nor disagree | Agree somewhat | Agree | Agree strongly | Do not know |
| --- | --- | --- | --- | --- | --- | --- | --- | --- | --- | --- |
| The relevant staff considered the situation and managed it so that I could keep working while receiving treatment | | | | | 1 | 2 | 3 | 4 | 5 | 6 |

1. **Please tell us about the steps you took to balance your treatment and work if applicable? (Choose any that apply)**
2. I spoke with my workplace HR consultant
3. Hourly and/or half day leave (taken on a regular or irregular basis)
4. Flexible working (starting early and/or finishing late)
5. Part-time working or shorter working hours
6. Working from home/working remotely
7. Trial working (for those who have been on leave for an extended period, working for a certain period of time or days when returning to work)
8. Others ()
9. None of above
10. **Before starting your treatment, did you receive any advice from the medical staff about continuing to work? (Choose one)**
11. Yes
12. No

Did you need any advice? (Choose one)

b1. Yes

b2. No

1. I do not know
2. **What has your work status been since you received your first cancer treatment?**

**(1) Regarding your employment, did you experience any of the following after your cancer diagnosis? (Choose one)**

1. I took leave of absence but did not resign or close my business → Please answer sub-question (2) below and then go to question 28.
2. I resigned or closed my business → please go to sub-question (3) below.
3. I did not experience any of the above → please go to question 28.
4. I do not know → please go to question 28.

**(2) This is a question for those who took leave of absence. Please tell us about your type of leave. (Choose any that apply)**

1. Paid leave
2. I received financial support other than paid leave (e.g., holiday pay, sick pay, and critical illness insurance cover)
3. Unpaid time off
4. Others ()

**What did you do after that? (Choose one)**

1. I went back to the previous job (at least once)
2. I never returned to work

**(3) This is a question for those who resigned or closed their business. When did that happen? (Choose one)**

1. When I was suspected of having cancer but before I was diagnosed
2. Shortly after I was diagnosed
3. After diagnosis but before the first treatment
4. During the first treatment
5. After the first treatment but before I was supposed to be back at work
6. After I returned to work
7. Others ()

**What did you do after that? (Choose one)**

1. I returned to the previous company or started a job at another company
2. I would like to return to work but I am currently unemployed
3. I have no wish to return to work

**If you do not mind, please tell us the reasons you resigned or closed your business in the Additional Comments section on p.19.**

**The following questions concern the provision of cancer treatment in general.**

1. **Please choose one response to each of the statements below**

|  |  |  |  |  | Disagree | Neither agree nor disagree | Agree somewhat | Agree | Agree strongly |
| --- | --- | --- | --- | --- | --- | --- | --- | --- | --- |
| 1. | Cancer treatment for the general public has improved compared to a few years ago. | | | | 1 | 2 | 3 | 4 | 5 |
| 2. | There is sufficient support, services and places for cancer patients and their families to discuss their concerns about cancer. | | | | 1 | 2 | 3 | 4 | 5 |

1. **Are you aware of the Cancer Counseling and Support Centers*? (Choose a or b)**
2. Yes

Have you ever used a Cancer Counseling and Support Center? (Choose one)

(1) No

Please tell us the reasons you did not use this service (Choose any that apply)

a1. I did not know about it when I needed it

a2. I did not want to discuss anything

a3. I did not know what services it provided

a4. I was concerned about confidentiality

a5. I was not confident that they would understand my concerns

a6. I was worried how other patients would perceive me

a7. Others ()

(2) Yes

How useful did you find the Cancer Counseling and Support Center? (Choose one)

a1. Very helpful

a2. Somewhat helpful

a3. A little helpful

a4. Neither helpful nor unhelpful

a5. Not helpful

1. No

**These Centers are nationwide and established in designated cancer hospitals.*

1. **Are you aware of peer support*? (Choose a or b)**
2. Yes

Have you ever used peer support? (Choose one)

(1) No

Please tell us the reasons why you did not use this (Choose any that apply)

a1. I did not know about it when I needed it

a2. I did not want to discuss anything

a3. I did not know what it entailed

a4. I was concerned about confidentiality

a5. I was not confident that they would understand my concerns

a6. I was worried how other patients would perceive me

a7. Others ()

(2) Yes

How useful did you find the peer support? (Choose one)

a1. Very helpful

a2. Somewhat helpful

a3. A little helpful

a4. Neither helpful nor unhelpful

a5. Not helpful

1. No

**This is where patients and survivors share their experiences and receive support from each other.*

1. **Do you know what clinical trials* are? (Choose one)**
2. Yes, I am very familiar with them
3. I am somewhat familiar with them
4. I have heard about them but am not particularly familiar with them
5. I have never heard of them

**These are targeted studies involving human trials that examine the efficacy and safety of drugs and medicinal devices.*

1. **Are you aware of any genome-based cancer treatments*? (Choose one)**
2. Yes, I am very familiar with them
3. I am somewhat familiar with them
4. I have heard about them but am not particularly familiar with them
5. I have never heard about them

**These are types of treatment based on medical research that has identified genetic abnormalities in cancer cells*

1. **Please read the following sentences and consider how they apply to your current situation. (Choose one answer for each question, ranking it between 1 and 5).**

|  |  |  |  |  | Disagree | Neither agree nor disagree | Agree somewhat | Agree | Agree strongly |
| --- | --- | --- | --- | --- | --- | --- | --- | --- | --- |
| 1. | I feel that because of my cancer I am a burden on my family | | | | 1 | 2 | 3 | 4 | 5 |
| 2. | I feel that because of my cancer I am a burden on people outside of my family | | | | 1 | 2 | 3 | 4 | 5 |
| 3. | I feel that I received too much unnecessary attention after I was diagnosed with cancer | | | | 1 | 2 | 3 | 4 | 5 |
| 4. | I feel discriminated against by people outside of my family because I have cancer | | | | 1 | 2 | 3 | 4 | 5 |
| 5. | I am able to consult with a medical professional when I feel pain or discomfort | | | | 1 | 2 | 3 | 4 | 5 |
| 6. | I am able to consult with a medical professional when I am experiencing mental distress | | | | 1 | 2 | 3 | 4 | 5 |
| 7. | I feel that I am able to go about my daily life now | | | | 1 | 2 | 3 | 4 | 5 |

1. **Please read the following sentences and consider how they apply to your current situation. (Choose one answer for each question, ranking it between 1 and 5)**

**PLEASE NOTE: The scale for these questions differs from that for previous questions**

|  |  |  |  |  | Disagree strongly | Disagree | Neither agree nor disagree | Agree | Agree strongly |
| --- | --- | --- | --- | --- | --- | --- | --- | --- | --- |
| 1. | I have sufficient support to relieve my physical pain and mental distress. | | | | 1 | 2 | 3 | 4 | 5 |
| 2. | I have physical pain caused by the cancer or the cancer treatment (including but not limited to nausea, shortness of breath, numbness, lethargy, itchiness and other physical distress) | | | | 1 | 2 | 3 | 4 | 5 |
| 3. | I have pain caused by the cancer or the cancer treatment. | | | | 1 | 2 | 3 | 4 | 5 |
| 4. | I have mental distress due to the cancer or the cancer treatment. | | | | 1 | 2 | 3 | 4 | 5 |
| 5. | Due to the pain and discomfort from the cancer or cancer treatment I have difficulties going about my daily life | | | | 1 | 2 | 3 | 4 | 5 |

**This is the end of the questionnaire.**

Please state the time at which you completed this questionnaire.

Finish time: _________________

**Thank you very much for your help and taking the time to complete this survey. Please feel free to write any further comments in the box below.**

**Additional comments**
